# Circulating tumor cell gene expression and plasma *AR* gene copy number as biomarkers for castration-resistant prostate cancer patients treated with cabazitaxel

**DOI:** 10.1186/s12916-022-02244-0

**Published:** 2022-01-31

**Authors:** Giorgia Gurioli, Vincenza Conteduca, Nicole Brighi, Emanuela Scarpi, Umberto Basso, Giuseppe Fornarini, Alessandra Mosca, Maurizio Nicodemo, Giuseppe Luigi Banna, Cristian Lolli, Giuseppe Schepisi, Giorgia Ravaglia, Isabella Bondi, Paola Ulivi, Ugo De Giorgi

**Affiliations:** 1 Biosciences Laboratory, IRCCS Istituto Romagnolo per lo Studio dei Tumori (IRST) “Dino Amadori”, Meldola, Italy; 2 Department of Medical Oncology, IRCCS Istituto Romagnolo per lo Studio dei Tumori (IRST) “Dino Amadori”, Meldola, Italy; 3 Department of Medical and Surgical Sciences, Unit of Medical Oncology and Biomolecular Therapy, University of Foggia, Policlinico Riuniti, Foggia, Italy; 4 Unit of Biostatistics and Clinical Trials, IRCCS Istituto Romagnolo per lo Studio dei Tumori (IRST) “Dino Amadori”, Meldola, Italy; 5 Medical Oncology Unit 1, Department of Clinical and Experimental Oncology, Istituto Oncologico Veneto IOV IRCCS, Padova, Italy; 6 Medical Oncology Department, IRCCS Azienda Ospedaliera Universitaria San Martino - IST Istituto Nazionale per la Ricerca sul Cancro, Genova, Italy; 7 Multidisciplinary Oncology Outpatient Clinic, Candiolo Cancer Institute, FPO-IRCCS, Candiolo, Italy; 8 Medical Oncology, Ospedale Sacro Cuore don Calabria, Negrar, Verona, Italy; 9 Candiolo Cancer Institute, FPO-IRCCS, Candiolo, Turin, Italy

**Keywords:** mCRPC, Cabazitaxel, AR-V7, *AR* copy number, CTC

## Abstract

**Background:**

Cabazitaxel improves overall survival (OS) in metastatic castration-resistant prostate cancer (mCRPC) patients progressing after docetaxel. In this prospective study, we evaluated the prognostic role of CTC gene expression on cabazitaxel-treated patients and its association with plasma androgen receptor (*AR*) copy number (CN).

**Methods:**

Patients receiving cabazitaxel 20 or 25 mg/sqm for mCRPC were enrolled. Digital PCR was performed to assess plasma *AR* CN status. CTC enrichment was assessed using the AdnaTest EMT-2/StemCell kit. CTC expression analyses were performed for 17 genes. Data are expressed as hazard ratio (HR) or odds ratio (OR) and 95% CI.

**Results:**

Seventy-four patients were fully evaluable. CTC expression of AR-V7 (HR=2.52, 1.24–5.12, *p*=0.011), AKR1C3 (HR=2.01, 1.06–3.81, *p*=0.031), AR (HR=2.70, 1.46–5.01, *p*=0.002), EPCAM (HR=3.75, 2.10–6.71, *p*< 0.0001), PSMA (HR=2.09, 1.19–3.66, *p*=0.01), MDK (HR=3.35, 1.83–6.13, *p*< 0.0001), and HPRT1 (HR=2.46, 1.44–4.18, *p*=0.0009) was significantly associated with OS. ALDH1 (OR=5.50, 0.97–31.22, *p*=0.05), AR (OR=8.71, 2.32–32.25, *p*=0.001), EPCAM (OR=7.26, 1.47–35.73, *p*=0.015), PSMA (OR=3.86, 1.10–13.50, *p*=0.035), MDK (OR=6.84, 1.87–24.98, *p*=0.004), and HPRT1 (OR=7.41, 1.82–30.19, *p*=0.005) expression was associated with early PD. *AR* CN status was significantly correlated with AR-V7 (*p*=0.05), EPCAM (*p*=0.02), and MDK (*p*=0.002) expression. In multivariable model, EPCAM and HPRT1 CTC expression, plasma *AR* CN gain, ECOG PS=2, and liver metastases and PSA were independently associated with poorer OS. In patients treated with cabazitaxel 20 mg/sqm, median OS was shorter in AR-V7 positive than negative patients (6.6 versus 14 months, HR=3.46, 1.47–8.17], *p*=0.004).

**Conclusions:**

Baseline CTC biomarkers may be prognosticators for cabazitaxel-treated mCRPC patients. Cabazitaxel at lower (20 mg/sqm) dose was associated with poorer outcomes in AR-V7 positive patients compared to AR-V7 negative patients in a post hoc subgroup analysis.

**Trial registration:**

Clinicaltrials.govNCT03381326. Retrospectively registered on 18 December 2017.

**Supplementary Information:**

The online version contains supplementary material available at 10.1186/s12916-022-02244-0.

## Background

Prostate cancer (PCa) is one of the leading causes of cancer death in men worldwide [1]. Androgen-deprivation therapy (ADT) is the cornerstone of treatment for advanced PCa until progression of disease (PD) to castration-resistant prostate cancer (CRPC). The Androgen receptor (AR) pathway is the main actor and *AR* mutations, *AR* amplification, constitutively active AR variants, intracrine steroid synthesis, and AR bypassing seem to be the mechanisms involved in CRPC evolution [2]. As the optimal treatment sequencing is still unclear and many novel agents are being investigated and progressively applied to clinical practice [3, 4], chemotherapy with docetaxel and cabazitaxel is preferred in patients with rapidly progressing disease, poor response to initial ADT, or presence of visceral metastases. Docetaxel is a taxane that was associated with improved overall survival (OS) in metastatic CRPC (mCRPC) [5]. However, mCRPC eventually develops resistance to docetaxel during treatment. Cabazitaxel is a next-generation tubulin-binding taxane, that demonstrated to improve OS in mCRPC patients progressing after docetaxel [6]. Taxanes seem to be able to overcome different mechanisms of resistance to androgen-signaling-targeted inhibitors, such as increased AR signaling [7–9]. This suggests that other mechanisms of resistance occur in taxanes-treated patients. Although serum prostate-specific antigen (PSA) is a biomarker for the assessment of therapeutic response and PD, it lacks specificity and has a limited prognostic and predictive value [10, 11]. Currently, there is a need for biomarkers to predict outcomes to cabazitaxel-treated mCRPC, in order to achieve a better selection of potentially responsive patients, maximize benefits, and avoid unnecessary treated-related adverse events.

Cell-free DNA (cfDNA) biomarkers, such as *AR* copy number (CN) have already been demonstrated to be useful liquid biopsy-based approaches, found to be associated with worse outcomes to AR-targeted therapies abiraterone and enzalutamide [12–14].

Circulating tumor cells (CTC) are released into the bloodstream from the primary tumor and/or metastasis. So, CTC analysis can be considered a non-invasive liquid biopsy test that takes into account the heterogeneity typical of PCa. Specific genomic alterations found in CTC are associated with clonal evolution or selection of cells contributing to treatment resistance in mCRPC [15]. Several studies demonstrated that patients with detectable CTC have a worse prognosis than those without [16, 17]. Therefore, gene expression profiles deriving from CTC could help to establish novel predicting biomarkers for PCa treatments. One of the most studied CTC-based biomarkers in PCa is the AR splice variant 7 (AR-V7) that is the best described and most abundant AR splice variant characterized by the lack of the C-terminal androgen-binding domain, making it constitutively active as a transcription factor, regardless of androgen signaling [18, 19]. In this study, we evaluated the prognostic role of a panel of CTC-expression biomarkers in mCRPC patients treated with cabazitaxel. We selected an immunomagnetic-based test for CTC enrichment and isolation able to identify epithelial-to-mesenchymal transition (EMT) that could instead be missed by epithelial-based CTC detection methods, such as CellSearch assay. EMT plays an important role in the metastatic process causing downregulation of epithelial proteins [20]. Moreover, stem cell-like tumor cells are considered a source of metastatic spread and have been identified within the population of CTC [21, 22]. This CTC isolation kit targeted EPCAM, EGFR, ERBB2: EPCAM expression is present on the surface of epithelial cells and it plays an important role in prostate cancer proliferation, invasion, metastasis [23]; EGFR expression is observed in CTC during prostate cancer metastasis and it promotes survival of prostate tumor-initiating cells and CTC that metastasize to bone; ERBB2 expression is elevated in bone metastases of prostate cancer. Moreover, aberrant activities of ERBB2 and EGFR have also been associated with the development of CRPC [24–26]. Finally, we explored the association between the expression of CTC-biomarkers and plasma *AR* CN status.

## Methods

### Aim and patient cohort

This prospective study (NCT03381326) was approved by the Institutional Review Board of IRCCS Istituto Romagnolo per lo Studio dei Tumori (IRST) “Dino Amadori,” Meldola, Italy (protocol code: IRST B030). Inclusion criteria were: histology of prostate adenocarcinoma without neuroendocrine differentiation, a progressive disease despite “castration levels” of serum testosterone (< 50 ng/dL), ongoing LHRH analog treatment or prior surgical castration, and evidence of PD during or after treatment with docetaxel. Additional eligibility criteria included an Eastern Cooperative Oncology Group (ECOG) performance status 0–2, adequate cardiac, renal, hepatic, and bone marrow function. PD was defined as either biochemical progression (three consecutive rises in PSA 1 week apart, or radiologic progression (consisting in the appearance of new lesions using the Response Evaluation Criteria in Solid Tumors) [10]. Serum PSA was evaluated within 3 days of beginning therapy and monthly thereafter. Radiographic disease was assessed with the use of computed tomography and bone scan at the time of screening and every 12 weeks on treatment. The study was conducted in accordance with the Declaration of Helsinki and the Good Clinical Practice guidelines of the International Conference of Harmonization. Written informed consent was obtained from all patients.

### Study design and blood sampling

We collected blood samples from 100 patients at different time points: pre-treatment, after one cycle treatment (optional), at first radiological evaluation, at the end of treatment/at PD. Peripheral blood samples were collected pre-treatment (baseline) in cfDNA BCT tubes (STRECK, USA), centrifuged at 1800×*g* for 15 min and plasma aliquots were stored at -80 °C. Two 5-ml peripheral blood samples from each patient were retrieved at different time points in AdnaCollect^TM^ tubes (Qiagen) to enrich and isolate CTC.

### DNA isolation and quantification

Plasma DNA was extracted with the QIAamp Circulating Nucleic Acid Kit (Qiagen, Milan, Italy) according to the manufacturer’s instructions, using 1 mL of plasma. Total extracted DNA was quantified by a spectrophotometer (Nanodrop ND-1000, Celbio, Milan, Italy) using 2 μl of DNA.

### Digital PCR analysis

*AR* copy number (CN) analyses were performed by QuantStudio3D digital Polymerase Chain Reaction (dPCR) System (Thermo Fisher Scientific) in a duplex assay using FAM and VIC fluorescent probes. *AR* CN was evaluated with two assays (AR1: Hs04107225; AR2: Hs04511283) and two reference genes were selected as control genes: *RNaseP*, TaqMan Copy Number Reference Assay, and *AGO1* (Hs02320401), modified with a VIC-labeled probe. DNA samples from three healthy male donors were pooled and used as calibrator. Data were analyzed using QUANT STUDIO 3D ANALYSIS SUITE CLOUD Software (Thermo Fisher Scientific). The average number of copies per reaction microlitres was determined using Poisson distribution. A ratio of target copies and reference copies was measured for each sample, then a ratio between sample and calibrator was calculated. *AR* CN status is defined as the mean value of the result of the two assays. AR gain status was defined using a cut-point value > 2.01, as previously described [14].

### CTC enrichment and detection

CTC were isolated by the immunomagnetic-based AdnaTest *EMT-2/StemCell Select*™, targeting *EPCAM*, *EGFR*, and *ERBB2*. This method permits the characterization of CTC and the assessment of potential biomarkers in CTC, even though it does not allow CTC enumeration or morphology.

The cell lysates derived were stored at − 20 °C no longer than 2 weeks, then proceeding with mRNA-isolation by AdnaTest *EMT-2/StemCell Detect*™ (both Qiagen) according to manufacturer’s instructions. Oligo (dT)25-coated beads allow mRNA isolation from the lysate of pre-enriched CTC. cDNA was obtained from reverse transcription using Sensiscript Reverse Transcriptase Kit™ (Qiagen), as previously described [27].

### Gene expression profiling

#### List of target genes

We selected 17 cancer-related assays from the TATAA GrandPerformance CTC Assay Panel, a customizable gene panel that allows single-cell expression profiling that was run at TATAA Biocenter. Biomarkers’ choice was based on their role in prostate cancer progression and metastasis, steroid synthesis and signaling, stemness, EMT process, neuroendocrine differentiation. All samples were profiled in duplicates for expression of AR-V7, AKR1C3, AKT2, ALDH1, AR, EPCAM, PSMA, MDK, PARP, MRP1, PI3KCA, POU5F1, PSCA, TUBB3, VIM, ACTB, and HPRT1. This gene expression panel is composed of 17 genes, of which ACTB was used as a control gene. The limit of quantification (LoQ) for the assays in the panel ranged from 20 to 200 copies/reaction. LoQ was calculated as the concentration of the last standard point before the RSDr (relative standard deviation of replicates) is > 35% based on copy number. Regarding specificity, capillary gel electrophoresis was performed during assays validation in order to identify that the PCR product had the correct length in bp (+/− 10 bp) as expected, indicating that the specific target was amplified.

CTC positivity for the examined patient cohort was defined as the expression of at least 1 of the following 12 biomarkers: AR-V7, AKR1C3, AKT2, ALDH1, AR, EPCAM, PSMA, MDK, PIK3CA, PSCA, TUBB3, VIM. These biomarkers were selected based on literature data supporting their capacity to identify CTC as positive in prostate cancer or confirming their role in prostate cancer or in EMT [28–30].

#### Preamplification

The samples were preamplified using TATAA PreAmp Primer Mix (TATAA Biocenter AB) and TATAA PreAmp GrandMaster® Mix (Cat. No. #TA05, TATAA Biocenter AB). Preamplification was performed in a thermocycler (T100, BioRad). No template controls (preAmp NTC) and human genomic DNA samples (0.5 ng/μl, TATAA Biocenter) were included.

#### qPCR

The preamplified samples were 8× diluted according to the recommendation for the TATAA PreAmp GrandMaster® Mix. The diluted preamplified samples were analyzed for the 17 genes selected from the GrandPerformance CTC Assay Panel (TATAA Biocenter AB) and the ValidPrime™ assay (TATAA Biocenter AB). The qPCR analysis was performed using TATAA Probe GrandMaster® Mix (TATAA Biocenter AB). No template control and preAmp NTC were included, as described elsewhere [31]. Cycle of quantification (Cq) values above 35 were treated as “off scale data” and replaced with a Cq of 50. The raw data (averaged Cq-values) were examined and corrected for genomic DNA contamination using the GenEx software (MultiD Analyses AB). The averaged Cq-values corrected with more than 1 Cq-value were removed from the analysis due to large-scale genomic DNA contamination. The expression data were normalized to the *ACTB* control gene for all the testing genes due to its stability and abundance in most of the samples analyzed. The Cq values were converted to relative quantities and transformed to log base 2 scale.

### Statistical analysis

Biomarker values evaluated at baseline and over time were summarized among all patients for whom at least one blood sample was available using descriptive statistics (absolute and relative frequency for categorical biomarkers whereas means ± standard deviation or median and interquartile range for continuous biomarkers).

Relationship among clinical characteristics and the value of each biomarker at baseline was investigated using chi-square test or Fisher’s test, as appropriate.

OS was calculated from the date of the start of cabazitaxel to death or last follow-up. Progression-free survival (PFS) was calculated as the time between the start of cabazitaxel and the first date of progression or death, whichever comes first, or last tumor evaluation.

Time-to-event outcomes (i.e. PFS and OS) were evaluated using the Kaplan-Meier method. Univariate and multivariable Cox regression models were used to estimate the hazard ratio (HR) and relative 95% confidence intervals (95% CI). To identify prognostic factors for OS and PFS, multivariable Cox regression model with stepwise backward elimination method were performed.

The effect of the interaction between AR-V7 expression and initial cabazitaxel dose on OS was evaluated using Cox regression model including AR-V7 expression, initial cabazitaxel dose, and initial dose-by-AR-V7 expression.

The impact of change in biomarkers at various landmark times on survival outcomes was assessed by landmark analyses. Patients with early PD/death before the landmark times were excluded. For these analyses, PFS and OS times were measured from the landmark times to these survival outcomes. The landmark times were at 1 month and 3 months of treatment.

Logistic regression analysis was performed to estimate odds ratio (OR) and 95% CI of the association between biomarkers and PSA response and early PD, defined as PD occurring within the first three months of treatment.

All tests were two sided, and *p*< 0.05 was considered significant. Statistical analysis was conducted using SAS Statistical Software release 9.4 (SAS Institute, Cary, NC, USA).

## Results

### Patient characteristics

**Fig. 1:**
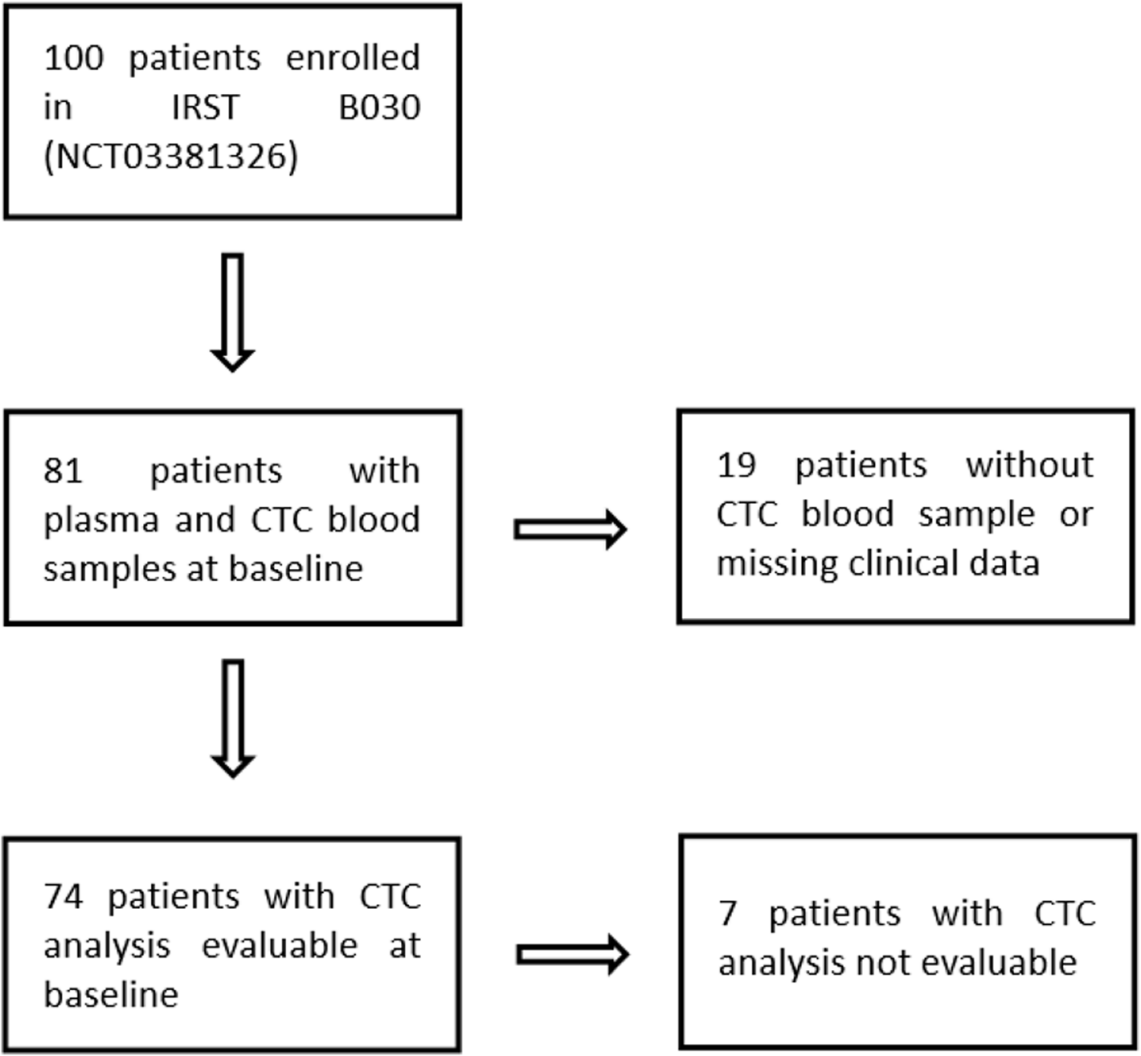
Flow chart of included and excluded patients

Between December 2014 and December 2018, 100 patients were recruited in the IRST B030 protocol. Among 100 patients enrolled, 74 were considered fully evaluable for CTC and *AR* CN analyses (Fig. 1). Cabazitaxel was administered with an initial reduced dose of 20 mg/sqm in 44 (59%) or with a full initial dose of 25 mg/sqm in 30 (41%) patients every 21 days, in both cases with prednisone 5 mg twice daily. Treatment dose was selected according to the physician’s choice based on the patient’s conditions. Treatment was administered for a maximum of ten cycles or until evidence of PD or unacceptable toxicity. All patients received prior treatment with docetaxel and 57 (77%) patients received also prior abiraterone or enzalutamide. Patient’s baseline characteristics are summarized in Table 1. Median age at enrollment was 72 years (range 49–82). Bone metastases were present in 72 (97%) cases and visceral metastases in 11 (15%); 6 (8%) patients presented liver metastases. Before enrollment, 17 (23%) patients had received one prior line of treatment, 43 (58%) two lines, 14 (19%) three lines. *AR* CN normal was found in 40 (54%) patients, whereas *AR* CN gain in 34 (46%). Median follow-up was 37 months (range 1–61). Median PFS and OS were 6.9 (95% CI 5.2–8.5) and 14.1 (95% CI 11.1–18.7) months, respectively. Univariate analysis reported that ECOG PS (*p* = 0.043 and *p* = 0.005), liver metastases (*p* = 0.005 and *p* = 0.0001), PSA (*p* = 0.0002 and *p* < 0.0001) and *AR* CN status (*p* = 0.0003 and *p* = 0.003) were significantly associated with PFS and OS, respectively. Gleason score (*p* = 0.01) and presence of pain (*p* = 0.028) were significantly associated only with PFS or OS, respectively.

**Table 1:** Patient characteristics

	***N*** (%)
**Patients enrolled and fully evaluable**	74 (100)
**Age**, median value (range, IQR)	72 (49–82, 67–77)
**ECOG Performance Status**	
0–1	55 (74.3)
2	19 (25.7)
**Gleason score**	
< 8	14 (22.2)
≥8	49 (77.8)
Unknown/missing	11
**Bone mts**	72 (97.3)
**Visceral mts**	11 (15.1)
**Liver mts**	6 (8.2)
**Lymph node mts**	42 (57.5)
**Presence of pain**	25 (36.8)
**Previous surgery** (radical prostatectomy)	33 (44.6)
**Previous radiotherapy** (radical radiotherapy)	32 (43.2)
**Number of previous treatments**	
1	17 (23)
2	43 (58.1)
3	14 (18.9)
***AR*** **CN**	
Normal	40 (54.0)
Gain	34 (46.0)
**Alkaline Phosphatase** U/l, median value (range, IQR)	174 (54–965, 101–270)
**Lactate Dehydrogenase** U/l, median value (range, IQR)	240 (76–1177, 181–345)
**Hemoglobin** g/l**,** median value (range, IQR)	12.0 (9.0–15.0, 10.9–13.1)
**Neutrophils count,** median value (range, IQR)	4150 (840–8730, 3100–5360)
**Lymphocytes count,** median value (range, IQR)	1495 (90–26,000, 1120–1900)
**Platelets count,** median value (range, IQR)	236,000 (115,000–719,000, 196,000–295,000)
**Serum Albumin** g/l**,** median value (range, IQR)	31.3 (3.7–42.0, 4.0–40.0)
**PSA**, median value (range, IQR)	54.56 (2.69–5000, 19–175.60)

*Abbreviations*: *IQR* interquartile range, *ECOG PS* Eastern Cooperative Oncology Group performance status, *mts* metastases, *AR* androgen receptor *CN* copy number *PSA* prostate-specific antigen

### Baseline CTC biomarkers and clinical outcome

At baseline, 67 of 74 (91%) samples were CTC positive (CTC+) and 7 out of 74 (9%) samples were classified as CTC negative (CTC−). After the first month of therapy, 20 out of 20 (100%) samples were CTC+. After the first radiological evaluation, 51 out of 56 (91%) samples were CTC+ and 5 out of 56 (9%) samples were CTC-. At the end of treatment, CTC were detected in 38 out of 43 samples (88%). Twenty-seven patients remained CTC+ over the whole observation period (only patients with pre-treatment, first radiological evaluation and end-of-treatment blood samples were considered). On the other hand, none of the patients remained CTC− at all the 3 time points evaluated. No association between CTC+/CTC− and clinical characteristics or outcome was found.

Correlation analysis of the biomarkers at baseline is shown in Additional file 1: Table S1. MRP1 expression was excluded because it was expressed in only 1 patient.

**Fig. 2:**
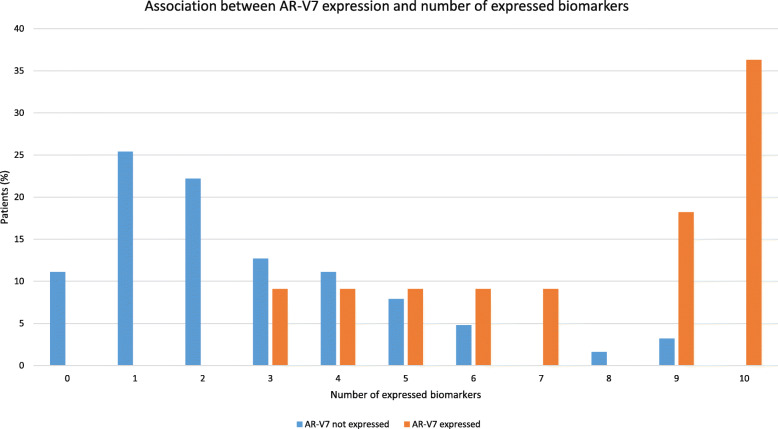
Association between AR-V7 expression and number of biomarkers. Histogram plot showing positive trend between the expression of AR-V7 (orange) and the co-expression of a growing number of other biomarkers (from 0 to 10, show in the *x*-axis)

**Fig. 3:**
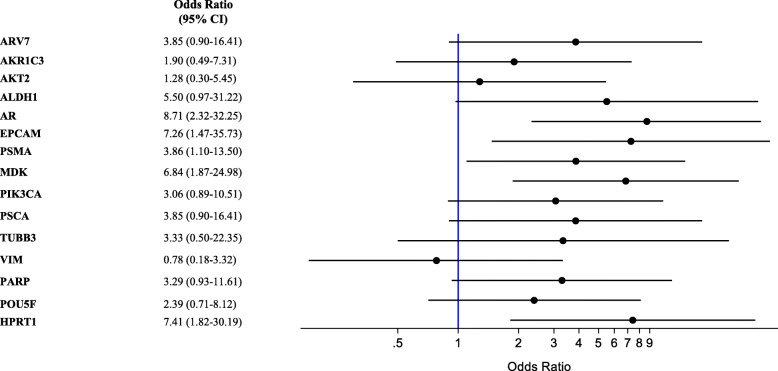
Correlation of CTC biomarkers and early PD. Forest plot showing the correlation between ALDH1, AR, EPCAM, PSMA, MDK, and HPRT1 CTC expression and early PD

CTC expression of AR-V7, AKR1C3, AR, EPCAM, PSMA, MDK, and HPRT1 was significantly associated with OS (Table 2). Moreover, we observed a trend between the expression of AR-V7 and the co-expression of a growing number of other biomarkers, as shown in Fig. 2. We found an association between ALDH1 (OR = 5.50, 95% CI 0.97–31.22, *p* = 0.05), AR (OR = 8.71, 95% CI 2.32–32.25, *p* = 0.001), EPCAM (OR = 7.26, 95% CI 1.47–35.73, *p* = 0.015), PSMA (OR = 3.86, 95% CI 1.10–13.50, *p* = 0.035), MDK (OR = 6.84, 95% CI 1.87–24.98, *p* = 0.004), and HPRT1 (OR = 7.41, 95% CI 1.82–30.19, *p* = 0.005) CTC expression and early PD (Fig. 3), defined as PD occurring within the first three months of treatment; whereas none of the CTC biomarkers was associated with PSA response (data not shown).

**Table 2:** Univariate analysis of overall survival in correlation with the biomarkers analyzed

	N. patients	N. events	Median OS (months) (95% CI)	*p* (logrank)	HR (95% CI)	*p* (Cox)
**AR-V7**						
Not expressed	63	48	16.4 (11.3–25.9)		1.00	
Expressed	11	10	10.6 (1.3–16.7)	0.008	2.52 (1.24–5.12)	0.011
**AKR1C3**						
Not expressed	58	45	16.7 (12.4–22.7)		1.00	
Expressed	16	13	9.6 (4.6–12.8)	0.028	2.01 (1.06–3.81)	0.031
**AKT2**						
Not expressed	60	45	15.2 (11.1–22.7)		1.00	
Expressed	14	13	12.6 (5.0–18.1)	0.134	1.61 (0.86–3.00)	0.138
**ALDH1**						
Not expressed	67	54	14.0 (11.1–18.3)		1.00	
Expressed	7	4	27.0 (1.3–nr)	0.428	0.66 (0.24–1.84)	0.432
**AR**						
Not expressed	56	43	18.1 (13.0–25.9)		1.00	
Expressed	18	15	7.6 (3.7–12.4)	0.001	2.70 (1.46–5.01)	0.002
**EPCAM**						
Not expressed	37	25	25.9 (16.4–39.1)		1.00	
Expressed	37	33	9.8 (6.8–12.4)	< 0.0001	3.75 (2.10–6.71)	< 0.0001
**PSMA**						
Not expressed	48	36	18.1 (13.3–28.0)		1.00	
Expressed	26	22	10.2 (7.2–13.0)	0.009	2.09 (1.19–3.66)	0.010
**MDK**						
Not expressed	54	40	18.3 (13.3–27.0)		1.00	
Expressed	20	18	8.3 (3.9–12.4)	< 0.0001	3.35 (1.83–6.13)	< 0.0001
**PIK3CA**						
Not expressed	50	39	16.4 (11.1–22.7)		1.00	
Expressed	24	19	12.8 (5.2–22.0)	0.337	1.31 (0.75–2.28)	0.339
**PSCA**						
Not expressed	64	50	15.2 (11.7–22.6)		1.00	
Expressed	10	8	5.7 (1.3–16.4)	0.088	1.92 (0.90–4.10)	0.092
**TUBB3**						
Not expressed	69	54	14.3 (11.3–22.0)		1.00	
Expressed	5	4	3.1 (1.3–nr)	0.146	2.10 (0.75–5.86)	0.155
**VIM**						
Not expressed	14	13	11.5 (3.9–25.9)		1.00	
Expressed	60	45	15.2 (11.1–22.6)	0.105	0.60 (0.32–1.12)	0.109
**PARP**						
Not expressed	56	43	16.7 (11.3–22.7)		1.00	
Expressed	18	15	10.5 (4.6–15.2)	0.112	1.61 (0.89–2.91)	0.117
**POU5F**						
Not expressed	47	38	15.2 (11.3–22.6)		1.00	
Expressed	27	20	12.8 (6.8–34.4)	0.757	1.09 (0.63–1.88)	0.758
**HPRT1**						
Not expressed	44	33	18.3 (14.1–28.0)		1.00	
Expressed	30	25	8.6 (3.9–12.4)	0.0006	2.46 (1.44–4.18)	0.0009

*Abbreviations*: *pts* patients, *OS* overall survival, *CI* confidence interval, *HR* hazard ratio, *nr* not reached

### The prognostic impact of AR-V7 expression in patients treated with reduced doses of cabazitaxel

As an exploratory analysis, we investigated the impact of AR-V7 expression on treatment outcome in 44 patients (59%) treated with an initial reduced dose (20 mg/sqm) of cabazitaxel. Among these patients, 8 (18%) had AR-V7 CTC expression. Regarding OS and PFS, no difference was observed between AR-V7 positive (3/30, 10%) and negative patients treated with full-dose cabazitaxel. However, in the initial reduced dose subgroup, AR-V7 positive patients had a worse median OS (6.6 versus 14 months, HR = 3.46, 95% CI 1.47–8.17, *p* = 0.004) compared to AR-V7 negative patients.

The interaction test involving AR-V7 expression effect in the initial reduced and full doses groups suggested that the correlation between AR-V7 expressions and worse outcome was significantly associated with the initial reduced dose of cabazitaxel for OS (*p* = 0.056).

### AR CN analysis and clinical outcome

Of the 74 patients evaluated, 40 had *AR* CN normal and 34 *AR* CN gain at baseline sample. We observed statistically significant shorter median PFS and OS in pre-treatment *AR* CN gain patients compared to *AR* CN normal patients (PFS 5.1 versus 9.7 months, HR = 2.68, 95% CI 1.58–4.56, *p* = 0.0003 and OS 11.1 versus 22.6 months, HR = 2.26, 95% CI 1.32–3.88, *p* = 0.003, respectively). *AR* CN status was significantly associated with AR-V7 expression (AR-V7 was expressed in 24% of patients with *AR* CN gain versus 7% of patients with *AR* CN normal, *p* = 0.05), EPCAM (expressed in 65% of patients with *AR* CN gain versus 37% of patients wih *AR* CN normal, *p* = 0.02), and MDK (expressed in 44% of patients with *AR* CN gain versus 12% of patients with *AR* CN normal, *p* = 0.002).

A Cox multivariable model to evaluate the association between OS and eight biomarkers (*AR* CN status and CTC expression of AR-V7, AKR1C3, AR, EPCAM, PSMA, MDK, and HPRT1) and four clinical prognostic factors such as ECOG PS, liver metastases, PSA, and presence of pain as covariates were constructed and analyzed in our cohort. The clinical prognostic factors selected have been demonstrated to be independent predictors of OS in univariate analysis in this cohort. Using Cox multivariable model with stepwise backward elimination method, EPCAM and HPRT1 CTC expression, plasma *AR* gain, ECOG PS = 2, presence of liver metastases and PSA were all independently associated with shorter OS (Table 3).

**Table 3:** Multivariate analysis of overall survival after stepwise backward elimination method^a^

	HR (95% CI)	*p*
**EPCAM**		
Not expressed	1.00	
Expressed	3.22 (1.68–6.17)	0.0004
**HPRT1**		
Not expressed	1.00	
Expressed	2.23 (1.22–4.07)	0.009
***AR*** **CN**		
Normal	1.00	
Gain	2.18 (1.22–3.87)	0.008
**ECOG PS**		
0–1	1.00	
2	2.85 (1.52–5.34)	0.001
**Liver mts**		
No	1.00	
Yes	3.30 (1.20–9.13)	0.021
**PSA** (continuous variable)	1.01 (1.00–1.01)	< 0.0001

*Abbreviations*: *HR* hazard ratio, *CI* confidence interval, *mts* metastases, *ECOG PS* Eastern Cooperative Oncology Group performance status, *PSA* prostate-specific antigen

^a^This method allowed us to insert all the independent statistically significant variables at univariate analysis into the model first. Each variable was deleted one at a time if they did not contribute to the regression equation. Variables were deleted based on their statistical contribution. After the application of the stepwise backward elimination method, only EPCAM, HPRT1, *AR* CN, ECOG PS, liver metastases, and PSA remained independent prognostic factors at multivariate analysis

### CTC biomarkers modulation during treatment with cabazitaxel and clinical outcome

We then evaluated the modulation of CTC biomarkers from baseline to one and three cycles after therapy, correlating it with patients’ prognosis. Twenty patients had available CTC analysis at baseline and after the first month of treatment; we found that an increased expression of AR-V7 was significantly correlated with PFS and OS (HR = 3.31, 95% CI 1.01–10.85, *p* = 0.048 and HR = 6.40, 95% CI 1.66–25.09, *p* = 0.007, respectively). However, it has to be noted that this result is referred to a patient only. Fifty-six patients had available CTC analysis at baseline and after 3 months of treatment, we observed that OS was associated with AKT2 increased expression (HR = 0.54, 95% CI 0.29–0.98, *p* = 0.044), considering a variation cut-off of 10% for the gene expression.

### AR-V7 expression and interpatient heterogeneity during treatment

We identified 11 AR-V7 positive patients at baseline sample (before cabazitaxel treatment) with median PFS and OS of 5.2 (95% CI 0.7-10.6) and 10.6 (95% CI 1.3-16.7) months, respectively. Three of these patients expressed AR-V7 at baseline and during treatment: one patient remained positive in each the time points analyzed (baseline, after 3 cycles and at the end of treatment) and had PFS and OS of 2.5 and 8.6 months, respectively; another patient remained positive from baseline to the sample collected after 3 cycles of treatment corresponding to PD sample (PFS and OS of 2.1 and 12.3 months); the last patient was positive at baseline, became negative after 3 cycles treatment and then returned positive at the end of treatment (PFS and OS of 16.7 months). Additional file 2: Fig. S1 shows AR-V7 expression trend from baseline to the end of treatment.

## Discussion

The molecular characterization of CTC from mCRPC patients treated with cabazitaxel provided through a panel of specific biomarkers, including genes related to PCa and taxanes chemotherapy, with a promising applicability as “liquid biopsy,” has been studied here. Many CTC subpopulations with different phenotypes circulate in the blood stream of metastatic patients, so the detection of multiple genes by molecular assays is crucial.

Our study, performed on a prospectively collected cohort of patients with mCRPC undergoing treatment with cabazitaxel, showed that the expression of specific CTC biomarkers is able to identify patients with worse prognosis and/or experiencing early PD.

A prognostic multivariable model was designed to evaluate the association between OS and the most significant clinical and biological markers found in our analysis. EPCAM and HPRT1 CTC expression, plasma *AR* CN gain, ECOG PS = 2, the presence of liver metastases, and PSA were all independently associated with poorer OS. EPCAM is a transmembrane glycoprotein that is consistently expressed by epithelial-derived tumor cells and is associated with poor clinical outcome [32]. EMT is characterized by the loss of epithelial markers, such as EPCAM, and by an increase of mesenchymal biomarkers, such as vimentin. However, co-expression of epithelial and mesenchymal biomarkers on CTC has also been reported [33, 34]. Surprisingly, we found that HPRT1 was significantly associated with shorter OS at univariate and multivariate analysis. Literature data reported controversial results regarding this biomarker, but recently published studies highlighted that it is not a suitable control for cancer-related experiments as it exhibits expression variability when comparing normal to malignant samples [35]. Moreover, HPRT1 has a higher statistically significant expression on prostate cancer cells and is significantly upregulated in a large proportion of prostate cancer tissue samples [36].

Notably, AR-V7 expression that actually represents the most clinically relevant CTC biomarker [37, 38] in mCRPC was correlated with *AR* CN gain, confirming the previous results [39]. AR-V7 association to taxanes resistance is still controversial. Although several studies indicated that AR-V7 CTC expression was correlated to resistance to abiraterone and enzalutamide but not to taxanes [8, 28, 40], some literature data reported that constitutively active AR-Vs, such as AR-V7, might affect taxane sensitivity, considering the inhibitory effect of taxanes on AR nuclear translocation by compromising its transcriptional activity [41–43]. In the present study, we found that AR-V7 expression was correlated to worse OS and we speculate that this may be due to prior lines of systemic treatment, comprising AR-directed agents and taxane chemotherapy. Indeed, taking into account that AR-V7 positivity is more frequent in patients pretreated with AR inhibitors [28], the number and sequence of prior treatments may have an important impact on AR-V7 expression. Moreover, we observed a trend between the expression of AR-V7 and the co-expression of a growing number of other biomarkers, suggesting that AR-V7 could be indicative of a higher disease aggressiveness, as previously reported [44, 45]. Our additional exploratory analysis aimed to explore the impact of AR-V7 CTC expression and initial cabazitaxel dose, supported by PROSELICA trial results [46]. We found that AR-V7 positive patients treated with initial reduced dose had a worse median OS compared to AR-V7 negative patients. These preliminary results could indicate that the response to cabazitaxel of AR-V7 positive clones is dose-dependent, similarly to what has been found for *AR* CN gain [9].

We also performed a CTC expression analysis during cabazitaxel treatment in a subgroup of patients and we showed that AKT2 increased expression after 3 months of treatment was correlated with a better OS. Literature data reported that AKT2 overexpression resulted in a significant decrease in migration, whereas AKT2 knockdown promoted migration of PC3 and DU145 PCa cells [47]. However, these results need to be validated in a larger case series. Previous findings have also reported that both baseline CTC count and CTC changes during chemotherapy or AR-directed treatments in mCRPC patients were more closely associated with patient survival than were PSA changes [48].

We recognize some limitations of our study, such as the relatively modest sample size. For this reason, we had to exclude MRP1 from the analyses because it was expressed in one patient only. A technical limitation was represented by our CTC isolation protocol that allows for simple and fast samples processing, but does not allow for CTC enumeration; furthermore, no cell morphological assessment could be performed. As a result, gene expression is not adjusted for the number of CTC and may be influenced by CTC quantity. In addition, we considered *AR* CN status, AR-V7 and AR expression, but other *AR* aberrations, such as *AR* mutations, were not included in the current analysis; thus a complete scenario regarding *AR* status and its clinical utility would be needed.

## Conclusions

In conclusion, our results suggest that CTC expression biomarkers, especially if evaluated before treatment initiation, could give important prognostic indications in mCRPC cabazitaxel-treated patients, and that AR-V7 positive patients could avoid an initial reduced dose. Moreover, this non-invasive, liquid-biopsy-based approach overcomes the need for tissue biopsies from metastases, still representing a major issue in PCa clinical management. The availability of predictive biomarkers could improve treatment selection, identifying the patients most likely to respond to therapy while avoiding to treat those with a low probability of response, reducing the potential treatment-related adverse events and the costs on the health care systems. Larger prospective multi-center clinical trials are warranted.

## Supplementary Information

**Additional file 1: Table S1.** Correlation analysis of gene expression biomarkers; we performed an analysis to evaluate the correlation of all the biomarkers considered.

**Additional file 2: Figure S1.** AR-V7 expression trend from baseline to disease progression for all AR-V7 positive patients or for patients divided in: non responders and responders. The lines represented the AR-V7 expression trend for all patients. Globally, AR-V7 expression was stable from baseline to the first radiological evaluation (3 months) in our patients, whereas we found a modulation of AR-V7 expression from 3 months to PD (at progression) in non responders who became AR-V7 positive at progression. One responder patient changed AR-V7 expression from positive at baseline to negative at 3 months.

**Additional file 3.** Raw data: Raw data biomarkers prostate cancer; the dataset used and analysed during the current study.

## Data Availability

The datasets used and/or analyzed during the current study are presented as Additional file 3: Raw data.

## Abbreviations

PCa: Prostate cancer
ADT: Androgen-deprivation therapy
PD: Progression of disease
CRPC: Castration-resistant prostate cancer
AR: Androgen receptor
OS: Overall survival
mCRPC: Metastatic castration-resistant prostate cancer
PSA: Prostate-specific antigen
cfDNA: Cell-free DNA
CN: Copy number
CTC: Circulating tumor cells
AR-V7: AR splice variant 7
EMT: Epithelial-to-mesenchymal transition
dPCR: Digital Polymerase Chain Reaction
Cq: Cycle of quantification
PFS: Progression-free survival
HR: Hazard ratio
CI: Confidence intervals
OR: Odds ratio
IQR: Interquartile range
ECOG PS: Eastern Cooperative Oncology Group performance status
mts: Metastases
CTC+: CTC positive
CTC-: CTC negative
pts: Patients
nr: Not reached
