# Supplementary material for: Circulating tumor cell gene expression and plasma AR gene copy number as biomarkers for castration-resistant prostate cancer patients treated with cabazitaxel
Source: BMC Med. 2022 Jan 31;20:48. doi: 10.1186/s12916-022-02244-0 (PMC8805338; doi:10.1186/s12916-022-02244-0)
Supplement: Supplementary file 1 — Additional file 1: Table S1. Correlation analysis of gene expression biomarkers; we performed an analysis to evaluate the correlation of all the biomarkers considered. [file 12916_2022_2244_MOESM1_ESM.docx]

**Additional file 1: Table S1.** Correlation analysis of gene expression biomarkers

|  | **AKR1C3** | **AKT2** | **ALDH1** | **AR** | **EPCAM** | **PSMA** | **MDK** | **PIK3CA** | **PSCA** | **TUBB3** | **VIM** | **PARP** | **POU5F** | **HPRT1** |
| --- | --- | --- | --- | --- | --- | --- | --- | --- | --- | --- | --- | --- | --- | --- |
|  | **r_s_ (p)** | | | | | | | | | | | | | |
| **AR-V7** | 0.20 (0.800) | 0.00 (1.00) | 1.00 (-) | 0.22 (0.533) | 0.02 (0.960) | 0.14 (0.701) | 0.46 (0.294) | -0.05 (0.911) | 0.80 (0.200) | 1.00 (<0.0001) | 0.53 (0.139 | 0.88 (0.042) | -0.11 (0.819) | 0.55 (0.083) |
| **AKR1C3** | - | 0.14 (0.787) | -0.40 (0.600) | 0.24 (0.570) | 0.43 (0.144) | 0.15 (0.650) | 0.61 (0.060) | 0.70 (0.036) | -0.10 (0.873) | -0.50 (0.667) | 0.04 (0.897 | -0.02 (0.960) | 0.58 (0.100) | 0.41 (0.191) |
| **AKT2** |  | - | 1.00 (<0.0001) | 0.90 (0.037) | 0.45 (0.187) | 0.36 (0.385) | -0.03 (0.957) | 0.07 (0.866) | 0.00 (1.000) | -1.00 (-) | 0.18 (0.532) | 0.77 (0.016) | -0.02 (0.955) | -0.25 (0.589) |
| **ALDH1** |  |  | - | 0.00 (1.000) | -0.80 (0.104) | -0.80 (0.104) | 0.40 (0.600) | 0.80 (0.104) | 1.00 (-) | 1.00 (-) | 0.26 (0.623) | 0.90 (0.037) | 0.80 (0.200) | 0.63 (0.367) |
| **AR** |  |  |  | - | 0.48 (0.051) | 0.34 (0.198) | 0.05 (0.880) | 0.13 (0.732) | 0.79 (0.036) | 0.80 (0.200) | 0.20 (0.467 | 0.10 (0.777) | 0.07 (0.832) | 0.34 (0.221) |
| **EPCAM** |  |  |  |  | - | 0.54 (0.011) | 0.03 (0.918) | 0.31 (0.240) | -0.05 (0.911) | -0.50 (0.391) | 0.29 (0.111) | -0.40 (0.181) | 0.24 (0.365) | 0.41 (0.062) |
| **PSMA** |  |  |  |  |  | - | 0.24 (0.381) | 0.15 (0.649) | -0.25 (0.548) | -0.80 (0.104) | 0.18 (0.411) | -0.75 (0.008) | 0.31 (0.288) | 0.26 (0.307) |
| **MDK** |  |  |  |  |  |  | - | 0.22 (0.484) | 0.21 (0.644) | 0.10 (0.873) | -0.08 (0.743) | -0.05 (0.898) | 0.24 (0.484) | 0.70 (0.007) |
| **PIK3CA** |  |  |  |  |  |  |  | - | 0.60 (0.285 | 0.60 (0.400) | 0.29 (0.182) | 0.08 (0.811) | 0.69 (0.007) | 0.27 (0.324) |
| **PSCA** |  |  |  |  |  |  |  |  | - | 0.70 (0.188) | 0.86 (0.014) | 0.48 (0.233) | 0.57 (0.139) | 0.40 (0.320) |
| **TUBB3** |  |  |  |  |  |  |  |  |  | - | 0.40 (0.600) | 0.90 (0.037) | -0.60 (0.400) | -0.80 (0.200) |
| **VIM** |  |  |  |  |  |  |  |  |  |  | - | -0.15 (0.573) | 0.45 (0.027) | 0.11 (0.598) |
| **PARP** |  |  |  |  |  |  |  |  |  |  |  | - | 0.12 (0.751) | 0.03 (0.931) |
| **POU5F** |  |  |  |  |  |  |  |  |  |  |  |  | - | 0.71 (0.0009) |
| **HPRT1** |  |  |  |  |  |  |  |  |  |  |  |  |  | - |
