# Supplementary figures and images for: Circulating tumor cell gene expression and plasma AR gene copy number as biomarkers for castration-resistant prostate cancer patients treated with cabazitaxel
Source: BMC Med. 2022 Jan 31;20:48. doi: 10.1186/s12916-022-02244-0 (PMC8805338; doi:10.1186/s12916-022-02244-0)

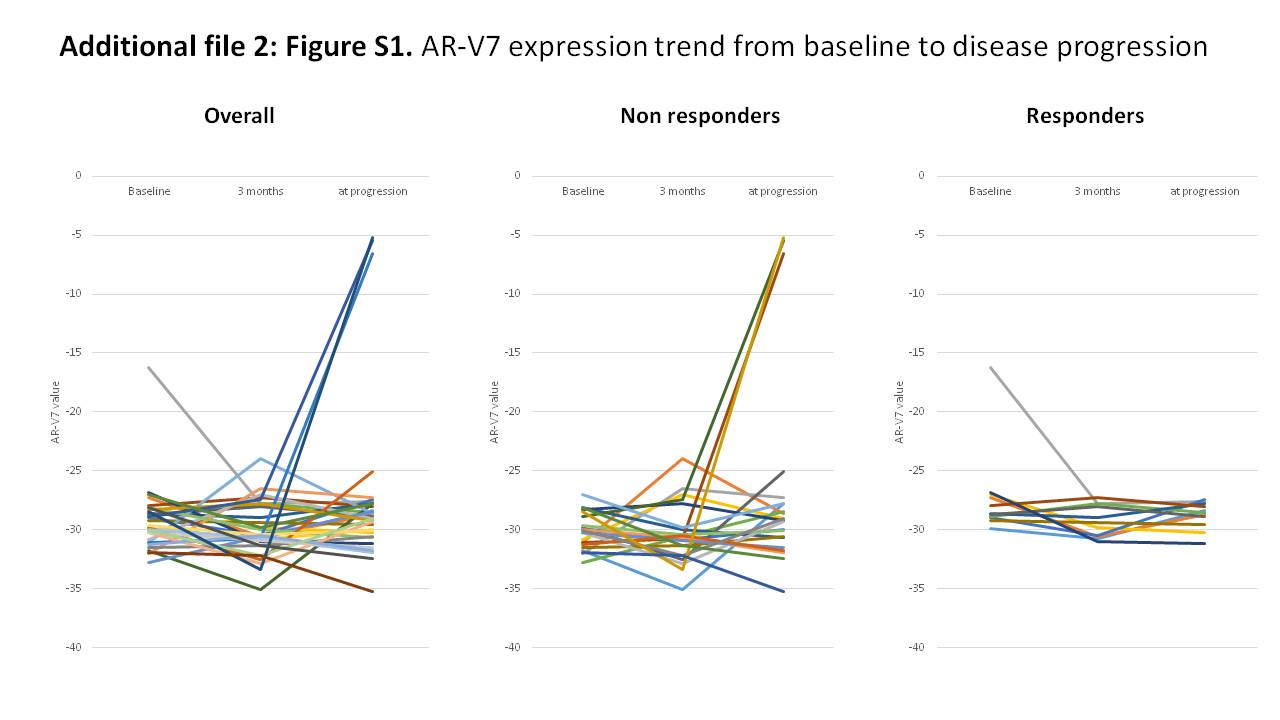

Supplement: Supplementary file 2 — Additional file 2: Figure S1. AR-V7 expression trend from baseline to disease progression for all AR-V7 positive patients or for patients divided in: non responders and responders. The lines represented the AR-V7 expression trend for all patients. Globally, AR-V7 expression was stable from baseline to the first radiological evaluation (3 months) in our patients, whereas we found a modulation of AR-V7 expression from 3 months to PD (at progression) in non responders who became AR-V7 positive at progression. One responder patient changed AR-V7 expression from positive at baseline to negative at 3 months. [file 12916_2022_2244_MOESM2_ESM.jpg]
